# Systemic Evaluation of the Effect of Diabetes Mellitus on Breast Cancer in a Mouse Model

**DOI:** 10.3389/fonc.2022.829798

**Published:** 2022-04-29

**Authors:** Nana Wei, Jinmiao Lu, Zhibing Lin, Xiaoyu Wang, Mengmeng Cai, Shengyao Jiang, Xiaoyu Chen, Shilan Zhu, Dong Zhang, Li Cui

**Affiliations:** ^1^ Shanghai Key Laboratory of Veterinary Biotechnology, School of Agriculture and Biology, Shanghai Jiao Tong University, Shanghai, China; ^2^ Key Laboratory of Adolescent Health Assessment and Exercise Intervention of Ministry of Education, East China Normal University, Shanghai, China; ^3^ Key Laboratory of Animal Parasitology of Ministry of Agriculture, Laboratory of Quality and Safety Risk Assessment for Animal Products on Biohazards (Shanghai) of Ministry of Agriculture, Shanghai Veterinary Research Institute, Chinese Academy of Agricultural Sciences, Shanghai, China

**Keywords:** diabetes mellitus, breast cancer, gut microbiome, tumor microenvironment, amino acid metabolism

## Abstract

Breast cancer complicated with diabetes mellitus (DM) is a common disease. To evaluate the effect of preexisting DM on breast cancer progression without drug interference, we used a streptozotocin (STZ)-induced type 2 diabetes mellitus BALB/c mouse model. We found that 4T1 breast cancer complicated with DM decreased the mouse survival time compared with 4T1-bearing mice. The diversity of gut microbiome was affected by DM. The infiltration of mucosal-associated invariant T cell (MAIT), CD8+ T cell, and CD4+ T cell in the tumor was significantly decreased in the DM-4T1 group compared with the 4T1 group. The transcriptome data of tumor tissues indicated that the expressions of inflammatory C–C chemokine- and metabolism-related genes were greatly changed. The abnormal expression of these genes may be related with the decreased T-cell infiltration in DM-4T1. In conclusion, the gut microbiome and tumor microenvironment of diabetic breast cancer patients have unique features. The effect of diabetes on breast cancer should be considered in the treatment for diabetic breast cancer patients.

## Introduction

Diabetes mellitus (DM) is a common chronic disease; 35% of the population will have a diagnosis of DM in their lifetime. There are many types of cancers complicated with diabetes mellitus, and 15% of the population will have diagnoses of cancers complicated with DM (1). Whether DM is a risk factor of the incidence and progression of cancer or not is a controversial issue. Most epidemiologic studies support a relationship between DM and increased risk of cancer (2–4). There are epidemiologic studies giving the opposite conclusion between DM and prostate cancer (5–8). The cancer types, age, race, and drug can all affect the epidemiologic conclusion (9–11).

Breast cancer is the most common neoplasm with great mortality among women, the second leading cause of cancer deaths. According to epidemiologic studies, about 16% breast cancer patients have DM. Evidence supports that the preexisting DM is an independent risk factor of breast cancer incidence and progression (12–14). An Asian women-based study found that the history of DM was not associated with the risk of breast cancer (9). The drug used for breast cancer therapy is the predisposing factor of DM (15, 16). Metformin, a well-studied drug, has been used for both DM and breast cancer therapy (17–20). In clinical studies, the evaluation of the effect of preexisting DM on breast cancer incidence and progression is mainly dependent on a cohort study and meta-analysis. All the data are collected after DM and breast cancer drug interference (12, 15, 16). A different effect of drug interference can lead to conflicting conclusions.

The complicated interaction of cancer and DM promotes us to explore the linkers between DM and cancer (21). To evaluate the effect of preexisting DM on breast cancer progression without interference, we used a streptozotocin (STZ)-induced type 2 diabetes mellitus BALB/c mouse model. In this model, we systemically analyzed the gut microbiome, T-cell infiltration, and the transcriptome of tumor tissue with and without DM. We found that the gut microbiome was significantly changed in STZ-induced DM mice. The infiltration of CD8+, CD4+, and mucosal-associated invariant T-cell (MAIT) cells was also significantly decreased in the breast cancer complicated with DM group. The expression of inflammatory C–C chemokines in the tumor was influenced and may be associated with T-cell recruitment to the tumor site. The present study will provide more clues for the therapy of patients diagnosed with both DM and breast cancer.

## Materials and Methods

### Animal Model

Female BALB/c mice (4–6 weeks old) were purchased from Shanghai JieSiJie Laboratory Animals Co., Ltd. (Shanghai, China). All animal experiments were performed with permission from local animal ethics committees (IACUC approval number shvri-SZ-20200420-03). BALB/c mice were induced diabetic by intraperitoneal STZ injection at a dose of 130 mg/kg body weight with a 29-gauge needle. STZ (S0130, Sigma-Aldrich, Darmstadt, Germany) was dissolved in 0.05 M citrate buffer (pH 4.5). The control group was injected with citrate buffer. The blood glucose meter was used for the detection of the concentration of blood glucose and when the level ≥12 mmol/l of the mice was considered as DM (22, 23). The luciferase-expressing 4T1 (4T1-Luc) cell line was purchased from Shanghai Institutes for Biological Sciences, Chinese Academy of Sciences (Shanghai, China), and the cell line was preserved in our lab and maintained in RPMI 1640 (C11875500BT, Gibco, Carlsbad, CA) with 10% FBS (Australia origin) and 1 μg/ml puromycin (P8230, Solarbio, Beijing, China). The cell line was regularly tested for *Mycoplasma* contamination using MycoBlue Mycoplasma Detector Kit (D101, Vazyme, Nanjing, China) and must be verified negative before the following experiments. The tumor cells were collected and counted using the TC20™ automated cell counter (Bio-Rad, Hercules, CA, USA). Each mouse was given a single subcutaneous injection with 1 × 10^6^ 4T1-Luc cells. The control groups were injected with PBS. The tumor was measured using a digital caliper, and the tumor volumes were calculated using the formula: Tumor volume = (Width^2^ × Length)/2. *In vivo* imaging of 4T1-Luc subcutaneous mice was performed using the IVIS^®^ Spectrum *In Vivo* Imaging System (IVIS Spectrum, Waltham, MA, USA). D-Luciferin was purchased from Invitrogen. Each mouse was given 150 mg Luciferin/kg body weight by intraperitoneal injection using a 25 × 5/8-gauge needle. The fluorescence intensity of tumor was detected using the IVIS^®^ Spectrum *In Vivo* Imaging System.

### Fecal Sample Collection and 16s rRNA Sequencing

The samples were collected after the model was confirmed. DNA were extracted according to the manual instruction of TIANamp Stool DNA Kit (DP328, TIANGEN, Beijing, China). 16s sequencing was performed using the MiSeq platform (Illumina, San Diego, CA, USA). The 16S (variable region 4 [v4]) rRNA gene was amplified with the primer pair 338F (ACTCCTACGGGAGGCAGCA) and 806R (GGACTACHVGGGTWTCTAAT) with a single multiplex identifier (MID) and adaptors (24, 25). DADA2, Vsearch (v2.13.4_linux_x86_64), and Cutadapt (v2.3) were used in denoising and clustering for sequence data. The taxonomic analysis was performed in R using the packages ggraph and ggplot2. The alpha diversity indices Chao1, Faith’s PD, Good’s coverage, Shannon, Simpson, Pielou’s evenness, and Observed species were used. The principal coordinate analysis (PCoA) was used to evaluate the beta diversity. The hierarchical cluster analysis was performed in R using the packages vegan, ape, and ggtree. The LDA effect size (LEfSe) was performed in python using the package LEfSe. The ASV (amplicon sequence variant) Venn diagram was created in R using the package VennDiagram. The metagenomeSeq analysis was performed in R using the package metagenomeSeq.

### Flow Cytometry

The blood and tumor tissues were collected, and the corresponding single-cell suspensions were generated. The following reagents were used for the identification of CD8+ T cell, CD4+ T cell, and MAIT in the blood and tumor: anti-CD3-FITC (#561798), anti-CD4-PB450 (#562891), anti-CD8-PE (#553033), anti-CD3-APC-A750 (#557596), anti-TCR beta-APC-A700 (#560705), and MR1 (#361106) (26, 27); all these antibodies were purchased from Becton Dickinson (San Jose, CA, USA). Briefly, the cells were blocked using the isotype control antibodies. After washing, the fluorescence-conjugated antibodies were added into the single-cell suspensions and incubated for 30 min at room temperature. The cells were washed and resuspended. The labeled cells were determined using the CytoFLEX (Beckman Coulter, Brea, CA, USA), and data were analyzed using FlowJo cell analysis software (FlowJo, LLC, Ashland, OR).

### Sample Collection, Sequencing, and Data Analysis

Tumor tissues were dissected and quickly frozen with liquid nitrogen. Total RNA was extracted with RNAprep Pure Tissue Kit (DP431, TIANGEN, China) according to the manufacturer’s instruction. The cDNA libraries were constructed using the 5× FastKing-RT SuperMix (KR118, TIANGEN, China). The sequencing was performed on the HiSeq™ 2500 (Illumina) platform (28). Raw data were processed, and the sequencing reads were mapped to the reference genomes using HISAT2 (http://ccb.jhu.edu/software/hisat2/index.shtml). The identification of differentially expressed genes (DEGs) across samples was performed using the edgeR package. The functional classification and pathway analysis were performed using Gene Ontology (GO) and Kyoto Encyclopedia of Genes and Genomes (KEGG).

### RNA Extraction and Quantitative Real-Time PCR Experiments

Total RNA was extracted from tissues using TRIzol (15596-026, Invitrogen, Carlsbad, CA, USA). cDNA was synthesized using HiScript^®^ III 1st Strand cDNA Synthesis Kit with gDNA wiper (R312, Vazyme Biotech Co., Ltd., China) by following the manufacturer’s recommendations. Quantitative real-time PCR (qRT-PCR) was performed using Taq Pro Universal SYBR qPCR Master Mix (Q712, Vazyme Biotech Co., Ltd., China) for 30 s at 95°C, 40 cycles of 95°C for 10 s, and 60°C for 30 s. The relative expression levels of the target genes in each sample were evaluated using the 2^-△△Ct^ method. Mouse actin, a housekeeping gene, was used as internal control. The qRT-PCR primers used in this study are listed in **Table S1**.

### Indirect Immunofluorescence and Hematoxylin and Eosin Staining

The tumor tissue specimens collected from DM and DM-4T1 groups were fixed in neutral-buffered 10% formalin. The samples were prepared for histological examinations. The CD8+ and CD4+ T-cell infiltration was investigated by immunofluorescence (IF). The primary anti-CD4 and anti-CD8 monoclonal antibodies and the secondary antibodies FITC-Goat Anti-Rabbit IgG and Cy3-AffiniPure™ Goat Anti-Mouse IgG were purchased from Servicebio (Wuhan, China). The DNA-specific dye DAPI (GDP1024, Servicebio, China) was used to label nuclear DNA. The slides were stained with hematoxylin and eosin (HE).

### Statistical Analysis

Statistical analyses of microbiome were performed in R using the different packages, such as phyloseq36, vegan37, FSA38, and coin39. We used the *t*-test to conduct pairwise comparisons of tumor volume between different groups. The difference of 4T1 and DM-4T1 survival curve was analyzed using the log-rank (Mantel–Cox) test.

## Results

### Breast Cancer 4T1 Complicated With DM Increased the Mortality of Mice

**Figure 1:**
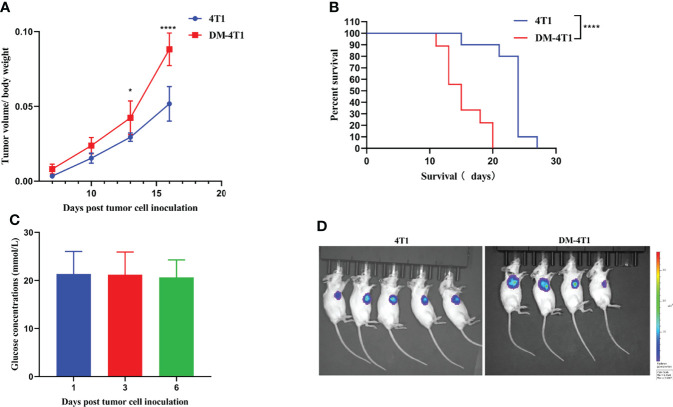
The preexisting diabetes mellitus leads to increased mortality rates in 4T1-bearing mice. **(A)** The ratio of tumor volume and body weight was calculated. **(B)** The survival curve of 4T1 and DM-4T1. **(C)** The glucose concentrations of DM mice. **(D)** *In vivo* imaging of the mice after exposure to the D-Luciferin 7 days post tumor cell inoculation. *p < 0.05; ****p < 0.0001.

To explore the role of DM in cancer progression, we developed an STZ-induced DM model without drug interference. We found that the tumor volume between breast cancer 4T1 (4T1) and breast cancer 4T1 complicated with DM (DM-4T1) had no significant difference. The ratio of tumor volume/mouse body weight in the DM-4T1 group was higher than that in the 4T1 group (**Figure 1A**). Compared with the 4T1 group, the survival time of mice in the DM-4T1 group was decreased (**Figure 1B**). The glucose concentration of STZ-induced mice was stable after tumor challenge (**Figure 1C**). There was no metastasis site found in both DM-4T1 and 4T1 groups, and the fluorescence intensity had no significant difference between the two groups (**Figure 1D**). These results showed that DM was a risk factor for 4T1 breast cancer; the tumor burden may cause the disease get worse or death in DM.

### DM Changed Gut Microbiota In 4T1 Tumor Bearing Mouse

**Figure 2:**
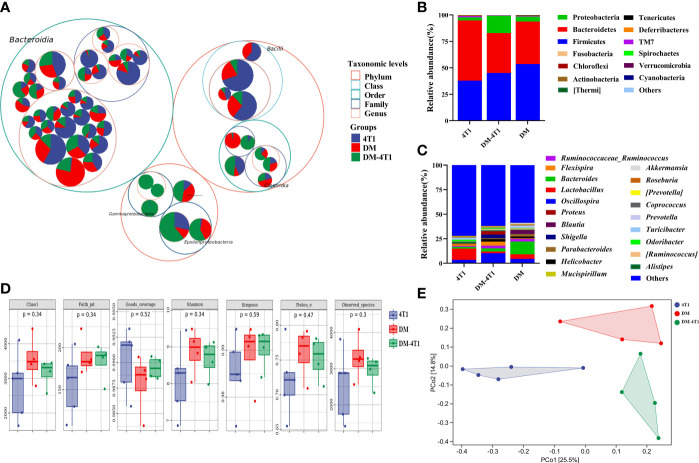
DM changed gut microbiome diversity. **(A)** Taxonomic differences are based on 16S rRNA sequencing. Taxonomic composition was visualized by circular packing. The largest circles represent phylum level, and the inner circles represent class, family, and genus, respectively. The circle sizes represent the mean relative abundance of the taxa. The taxa were colored by sample groups (red for DM-4T1 and blue for 4T1), whereas the area of the group corresponded to the mean relative abundance of the taxa in each group. **(B, C)** Total gut bacterial relative abundance at the taxonomic rank of phylum **(B)** and genus **(C)** of 4T1 and DM-4T1 groups. **(D)** Alpha diversity boxplot (Chao1, Faith pd, Goods coverage, Simpson, Pielou, and Observed_species) in 4T1 and DM-4T1 groups. **(E)** PCoA of microbial communities of 4T1 and DM-4T1. PCoA was calculated using the Bray–Curtis similarity measures.

**Figure 3:**
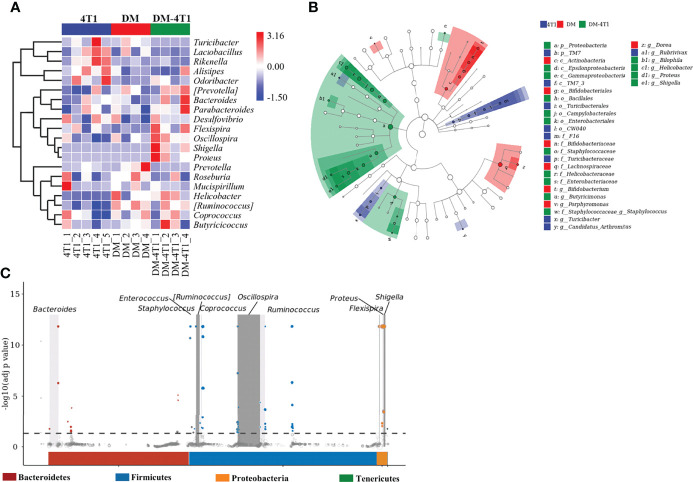
The difference and characteristics of bacteria in different groups. **(A)** The species composition heat map was created according to the Euclidean distance. **(B)** LEfSe comparison of gut microbiota between 4T1 and DM-4T1 groups. Taxonomic cladogram derived from LEfSe analysis of 16S sequences. (Red) DM-4T1-enriched taxa; (blue) taxa enriched in 4T1 group. **(C)** Manhattan plot. The x-axis represents the microbial ASV taxonomy at phylum level, and the y-axis represents -log10 (adj *p* value). Dots and hollow dots indicate ASVs with and without significant difference, respectively. The color of each marker represents the different taxonomic affiliation of the ASVs.

The gut microbiome has been reported to be changed on both cancer and DM development and progression (29–32). We found that breast cancer 4T1 complicated with DM increased the mortality in a mouse model (**Figure 1**). However, the causes or effects of the changed bacteria in gut of cancer complicated with DM are unclear. Therefore, in the present study, the gut microbiome differences among DM, 4T1, and DM-4T1 were determined. We found that DM could lead to microbiome imbalances. Bacteroidetes, Firmicutes, and Proteobacteria dominated the bacterial community (**Figure 2A**), and the bacterial composition between 4T1 and DM-4T1 showed differences at both the phylum and genus levels, and the differences were also present between DM and 4T1 and between DM and DM-4T1 groups (**Figures 2B, C**). Compared to 4T1, *Lactobacillus* and *Proteus* were significantly decreased and increased at the genus level, respectively, in the DM-4T1 group (**Figure 2C**). Different indexes were used to compare the alpha diversity, and there was no significant difference among the three groups (**Figure 2D** and **Table S2**). Furthermore, taxonomic beta diversity analysis was performed by PCoA based on the Bray–Curtis distance metrics. The PCoA diagram shows that the gut bacterial communities in 4T1 were distinct from the gut microbiome in DM and DM-4T1 (**Figure 2E**). A heat map representing the bacterial composition differences between individuals of different groups is displayed in **Figure 3A**. After data correction, the top 20 genera with different abundance could be found according to the color variations. Red represents the genus in high abundance, while blue represents low abundance. The bacterial composition of DM-4T1 could distinguish from that in the 4T1 and DM groups (**Figure 3B**). To determine the variations in gut microbiota composition and specific taxonomic biomarkers between the DM-4T1 and 4T1 groups, LEfSe and metagenomeSeq analyses were performed. LEfSe analysis showed that the relative abundance of bacterial taxa between the groups had significant difference (**Figure 3B**). MetagenomeSeq analysis showed that about 80 ASVs were significantly upregulated in DM-4T1, and these ASVs mainly belong to *Bacteroides*, *Staphylococcus*, *Enterococcus*, *[Ruminococcus]*, *Coprococcus*, *Oscillospira*, *Ruminococcus*, *Flexispira*, *Proteus*, and *Shigella* at the genus level (**Figure 3C**). These data indicated that DM changed the abundance and role of bacteria. Gut microbiome are associated with cancer immunotherapy. The DM-induced gut bacterial dysbiosis should be considered in the therapy of cancer complicated with DM patients.

### DM Decreased the Infiltration of T Cells in Tumor

**Figure 4:**
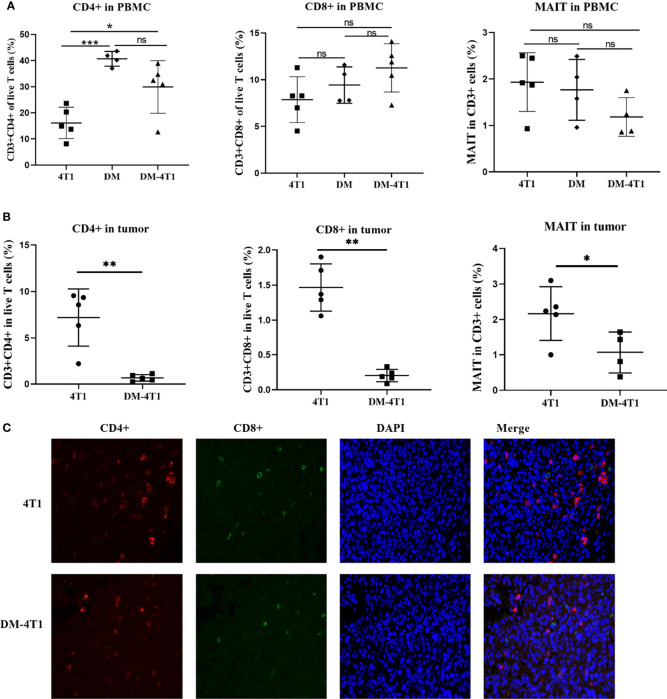
The infiltration of CD8+ T, CD4+ T, and MAIT cells were decreased in DM-4T1 group compared with 4T1 group. **(A, B)** The levels of CD8+ T, CD4+ T, and MAIT cells in PBMC **(A)** and in tumor **(B)** were detected using FCM. **(C)** The distribution of CD4+ T cell (red) and CD8+ T cell (green) in tumor tissue was confirmed using indirect immunofluorescence (IF). ns, no significant difference, *p ≤ 0.05; **p < 0.01;***p < 0.001.

The CD8+ and CD4+ T-cell infiltration was associated with the prognosis of cancer patients. The development and recruitment of mucosal-associated invariant T cells (MAIT) depends on the metabolites of bacteria. In this study, the level of CD4+ T cells in PBMC and in tumor between different groups was determined using flow cytometry (FCM). Gating strategies are shown in **Figures S1A, B**. The results showed that DM could affect the CD4+ T-cell level in PBMC, compared to the 4T1 group, but not the DM-4T1 group (**Figure 4A**). Furthermore, we found that the level of CD4+ T cells in PBMC in the DM-4T1 group was increased compared with that in the 4T1 group (**Figures 4A**, **S1C**), while the levels of CD8+ and MAIT T cells in PBMC had no significant difference between the 4T1 and DM-4T1 groups (**Figures 4A**, **S1D, E**). Furthermore, we found that the levels of CD4+ T cells, CD8+ T cells, and MAIT cells in tumor were significantly decreased in the DM-4T1 group, compared to the 4T1 group (**Figures 4B**, **S1C–E**). IF was also used to analyze the level and distribution of CD4+ T cells and CD8+ T cells in tumor, and the decreased T-cell infiltration in DM-4T1 was further confirmed (**Figure 4C**), consistent with the results shown in **Figure 4B**.

### DM Affected the Transcriptional Response

**Figure 5:**
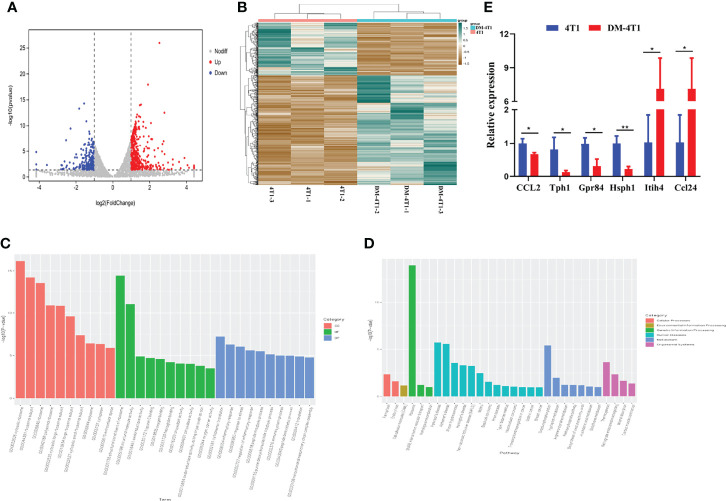
STZ-induced DM changed the transcriptome of tumor. **(A)** Volcano plot showed the changes of gene expression. The red dots indicate up regulation. The blue dots indicate downregulation. The gray dots indicate no significant difference. **(B)** The heat map of DM-4T1 versus 4T1 group. Transcript enrichment was encoded in the heat map from low (green) to high (red). The color bars at the top of the heat map indicated the clusters in two groups. **(C)** Top 10 GO categories were analyzed according to –log10 (*p* values). GOs included biological process (BP), cellular component (CC), and molecular function (MF). **(D)** A Bar diagram of the top 20 ranked KEGG pathways of DEGs. In the bar diagram, colors indicated different category. **(E)** Further validation of the RNA sequencing results using qRT-PCR. *p < 0.05, **p < 0.01.

The molecular mechanisms of breast cancer complicated with DM are unknown. We performed RNA sequencing, and the transcripts were annotated according to the known proteins in the UniProtKB/Swiss-Prot database. In total, 596 differentially expressed genes (DEGs) were identified between 4T1 and DM-4T1 (**Figure 5A**). The relative distribution of transcripts was clearly displayed by heat map, and the transcripts had a significant difference between the 4T1 and DM-4T1 groups (**Figure 5B**). GO and KEGG enrichment analyses implicated that these genes were involved in diverse processes, including cytosolic ribosome, structural constituent of ribosome, inflammatory response and cytoplasmic translation, arginine and proline metabolism, tryptophan metabolism, and pentose phosphate pathway (**Figures 5C, D**). Further, external validation of the genes using qRT-PCR was consistent with the results of RNA sequencing (**Figure 5E**).

## Discussion

The gut bacteria are considered as a real organ. The diversity of bacteria has greatly changed in cancer patients (33). Some bacterial species have been confirmed to play a key role in cancer development and progression, such as *Enterococcus faecalis*, *Clostridium septicum*, and *Helicobacter pylori* (34, 35). The blockade of immune checkpoints has been applied for cancer therapy. Recent studies found that the changed microbiome was associated with the immunotherapy efficacy (36). In our study, we found that both tumor and SZT-induced DM changed the gut microbiota diversity and abundance. In clinical gut microbiome studies, researchers focused on the changed bacteria and unique feature of microbiome. The interaction of host and gut bacteria was complicated, and the cause and effect are unclear. The changed bacterial species could be used as a diagnostic marker or cancer therapy (37, 38). The fecal microbiota transplantation (FCM) has been considered for the therapy of gut disease and infection (39, 40). However, the status of DM and cancer may affect and even impair the gut microbiota colonization in intestines. The causal links between changed bacterial species and diseases should be illustrated in future study.

The development and activation of MAIT cells were associated with bacterial metabolites (41). The infiltration of MAIT cells was associated with cancer initiation and growth in an MHC class I-like molecule (MR1)-dependent way (27). The MAIT infiltration was greatly decreased in the DM complicated with breast cancer group. The infiltration of CD8+ and CD4+ T cells was also decreased in the DM complicated with breast cancer group. T-cell infiltration was associated with prognosis of cancer patients. The T-cell recruitment to the tumor site was regulated by chemokines. The abnormal expression of C–C chemokines was reported and was associated with increased risk of type 2 diabetes mellitus (42, 43). The decreased infiltration of MAIT, CD8+, and CD4+ T cells may influence the effect of immunotherapy. In the treatment of cancer complicated with DM, the abnormal chemokine expression must be carefully evaluated and studied.

Both diabetes and cancer are complicated metabolic diseases. We explored the molecular basis of the link between breast cancer and diabetes through transcriptome. In this study, 596 DEGs were detected between the 4T1 and DM-4T1 groups. GO term enrichment analysis showed that these DGEs were involved in a variety of cellular processes, including ribosomes, extracellular space, cytoplasmic translation, inflammatory response, and immune system process. KEGG pathway enrichment analysis found that some DEGs were significantly enriched in ribosomes, oxidative phosphorylation, tryptophan metabolism, and thermogenesis (**Figure 4**). Recent years, many studies have demonstrated that oxidative phosphorylation is upregulated in a variety of cancers (44, 45), and oxidative phosphorylation is considered a potential target for cancer therapy (46). The function of oxidative phosphorylation involved in DM complicated with cancers needs to be further studied. In our present study, we found that heavy chain 4 (Itih4) was greatly increased in 4T1 complicated with DM (**Figure S1E**). The Itih family is a long-known family of serine protease inhibitors, which has been confirmed to be involved in various acute-phase processes, including inflammation or cancer (47). Itih4 has been confirmed to be an important marker associated with liver cancer (48, 49), early gastric cancer (50), and hepatocellular carcinoma diagnosis (51). However, the role of Itih4 in breast cancer remains unknown. Especially, there is no report about the role of Itih4 in breast cancer complicated with DM. In clinical studies, the function of Itih4 should be concerned. Previous studies showed that chemokine (C–C motif) ligand 24 (CCL24) is expressed in some tumor cells (52), and CCL24 was associated with cancer progression (53, 54). In our study, we found that DM could regulate CCL24 expression in cancer cells. It has been reported that CCL24 mainly has chemotactic activity for resting T lymphocytes, but not the activated T cells (55). Although we found the upregulation of CCL24 (**Figure 5E**) and the decrease in T-cell infiltration (**Figure 4**) in DM-4T1 in our study, the correlation between the CCL24 level and T-cell infiltration is not immediately clear and needs our further evaluation in the future study. According to the results, DM may promote cancer progression through inducing the abnormal expression of tumor-related genes; we suggest that the level of CCL24 should be evaluated in cancer patients complicated with DM. Other DEGs associated with metabolism and immunity, such as Tph1, Hsph1, and Gpr84, need to pay more attention in the future studies.

## Conclusion

In summary, we found that DM could change the gut microbiota and tumor microenvironment by either direct or indirect action. Therefore, we suggest that the gut microbiome and tumor microenvironment should be considered in the treatment of breast cancer complicated with DM. The results of our study may provide novel insights regarding potentially comprehensive therapies for breast cancer complicated with DM.

## Data Availability Statement

The original contributions presented in the study are included in the article/**Supplementary Material**. Further inquiries can be directed to the corresponding author.

## Ethics Statement

The animal study was reviewed and approved by the Institutional Animal Care and Use Committee of Shanghai Veterinary Research Institute (IACUC approval number shvri-SZ-20200420-03).

## Author Contributions

ZL and LC developed the study design and supervised the study. ZL, XW, JL, and MC mainly performed the mouse model and animal experiments. NW and XW performed the data analyses, data interpretation, and drafting of the manuscript. NW, SJ, XC, DZ, and SZ participated in the animal experiment, data collection, and data analysis. NW performed the experiment and the submission and revision of the manuscript. All authors contributed to the article and approved the submitted version.

## Funding

This work was supported by grants from the Shanghai Science and Technology Innovation Action Plan (21140901400, 21Z510202185) and the Shanghai Sailing Program (19YF1458600).

## Conflict of Interest

The authors declare that the research was conducted in the absence of any commercial or financial relationships that could be construed as a potential conflict of interest.

## Publisher’s Note

All claims expressed in this article are solely those of the authors and do not necessarily represent those of their affiliated organizations, or those of the publisher, the editors and the reviewers. Any product that may be evaluated in this article, or claim that may be made by its manufacturer, is not guaranteed or endorsed by the publisher.

## Supplementary Material

Supplementary Figure 1 The levels of CD3+CD4+, CD3+CD8+ T cells and MAIT in tumor and PBMC were evaluated using FCM. **(A)** Gating strategies for frequency analysis of CD3+CD4+ and CD3+CD8+ T cells. Cells were stained with anti-CD3-FITC, anti-CD4-PB450, anti-CD8-PE. **(B)** Gating strategies for frequency analysis of MAIT. Cells were stained with anti-CD3-APC-A750, anti-TCR beta-APC-A700, anti- MR1-PE. **(C)** CD3+CD4+ T cells in PBMC and in the tumor tissues of mice from 4T1 and DM-4T1 groups were stained and analyzed using FCM. **(D)** CD3+CD8+ T cells in PBMC and in the tumor tissues of mice from different groups were stained and analyzed using FCM. **(E)** MAIT in PBMC and in the tumor tissues of mice from different groups were stained and analyzed using FCM.
