# Supplementary figures and images for: Systemic Evaluation of the Effect of Diabetes Mellitus on Breast Cancer in a Mouse Model
Source: Front Oncol. 2022 Apr 29;12:829798. doi: 10.3389/fonc.2022.829798 (PMC9106558; doi:10.3389/fonc.2022.829798)

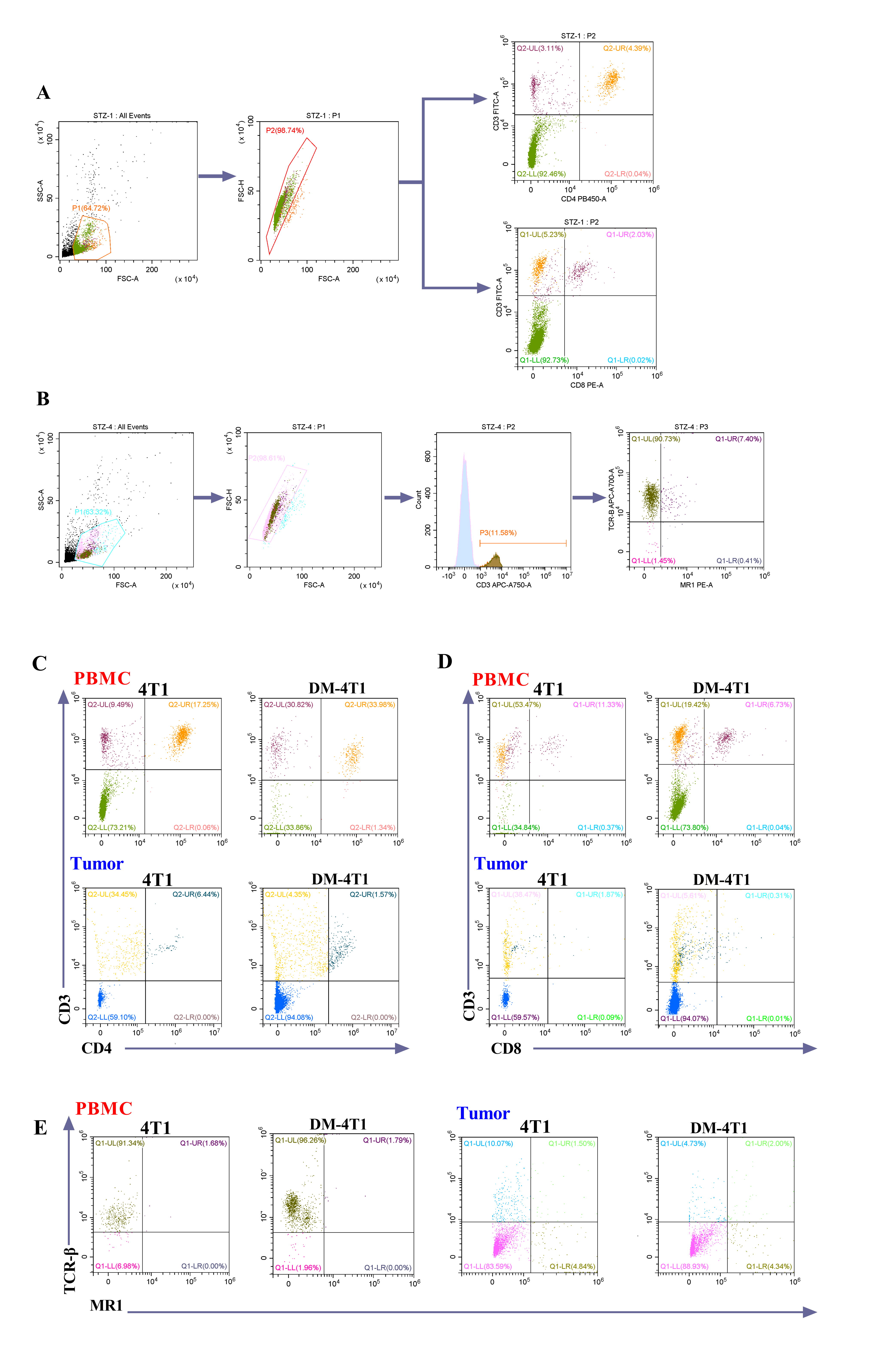

Supplement: Supplementary Figure 1 — The levels of CD3+CD4+, CD3+CD8+ T cells and MAIT in tumor and PBMC were evaluated using FCM. (A) Gating strategies for frequency analysis of CD3+CD4+ and CD3+CD8+ T cells. Cells were stained with anti-CD3-FITC, anti-CD4-PB450, anti-CD8-PE. (B) Gating strategies for frequency analysis of MAIT. Cells were stained with anti-CD3-APC-A750, anti-TCR beta-APC-A700, anti- MR1-PE. (C) CD3+CD4+ T cells in PBMC and in the tumor tissues of mice from 4T1 and DM-4T1 groups were stained and analyzed using FCM. (D) CD3+CD8+ T cells in PBMC and in the tumor tissues of mice from different groups were stained and analyzed using FCM. (E) MAIT in PBMC and in the tumor tissues of mice from different groups were stained and analyzed using FCM. [file Image_1.tif]
