# Supplementary material for: Systemic Evaluation of the Effect of Diabetes Mellitus on Breast Cancer in a Mouse Model
Source: Front Oncol. 2022 Apr 29;12:829798. doi: 10.3389/fonc.2022.829798 (PMC9106558; doi:10.3389/fonc.2022.829798)
Supplement: Supplementary file 2 [file Table_1.docx]

**Supplement table**

**Table S1** Primer sequences used in qRT-PCR analysis. Note: F means forward primer; R indicates reverse primer

| Gene name | Primer sequence (5’→3’) | Amplicon Size (bp) |
| --- | --- | --- |
| Gpr84-F | CTCCTGCTACCATGAGTCTGT | 186 |
| Gpr84-R | GTGCAGTAGAGTAGATCAGCCA |  |
| Tmem74-F | CCACTCTCTGTCTAAGAGGAACA | 174 |
| Tmem74-R | CTAAGTCTACACACTTCCACCAC |  |
| Tph1-F | AACAAAGACCATTCCTCCGAAAG | 119 |
| Tph1-R | TGTAACAGGCTCACATGATTCTC |  |
| Hsph1-F | GGGCTAGACGTAGGCTCACA | 281 |
| Hsph1-R | CCACCATTTTTCATTGGGACCA |  |
| Itih4-F | GTGGAACCTTGTGCTGTTCTT | 61 |
| Itih4-R | CTCGGCAGTAGTGGTCGGA |  |
| GcK-F | ATGGCTGTGGATACTACAAGGA | 137 |
| GcK-R | TTCAGGCCACGGTCCATCT |  |
| Actin-F | AGAGGGAAATCGTGCGTGAC | 195 |
| Actin-R | CCATACCCAAGAAGGAAGGCT |  |

**Table S2** **Alpha-diversity indexes in the gut of adults from different groups**

| Sample | Chao1 | Faith_pd | Goods_coverage | Observed_species | Pielou_e | Shannon | Simpson | Group |
| --- | --- | --- | --- | --- | --- | --- | --- | --- |
| 4T1_1 | 4105.57 | 204.772 | 0.985727 | 3815.1 | 0.794132 | 9.44819 | 0.994503 | 4T1 |
| 4T1_2 | 2994 | 164.476 | 0.991721 | 2883.9 | 0.73196 | 8.41301 | 0.989609 |  |
| 4T1_3 | 3150.95 | 178.38 | 0.988092 | 2878.1 | 0.721492 | 8.29059 | 0.984862 |  |
| 4T1_4 | 1628.67 | 106.899 | 0.993326 | 1410.1 | 0.654759 | 6.84981 | 0.973783 |  |
| 4T1_5 | 2009.21 | 129.79 | 0.992037 | 1742.7 | 0.699516 | 7.53175 | 0.983502 |  |
| STZ_1 | 3553.67 | 181.011 | 0.98839 | 3316.4 | 0.782065 | 9.14656 | 0.992823 | DM |
| STZ_2 | 2782.14 | 171.378 | 0.990551 | 2563.7 | 0.709269 | 8.03176 | 0.979144 |  |
| STZ_3 | 4432.52 | 222.229 | 0.983796 | 4094 | 0.791372 | 9.4959 | 0.994467 |  |
| STZ_4 | 3413.32 | 185.625 | 0.98924 | 3219.5 | 0.758553 | 8.83912 | 0.990889 |  |
| STZ_4T1_1 | 2561.83 | 151.744 | 0.990605 | 2330.2 | 0.712475 | 7.9699 | 0.984236 | DM-4T1 |
| STZ_4T1_2 | 3417.51 | 200.359 | 0.988461 | 3189.1 | 0.773205 | 8.99928 | 0.993364 |  |
| STZ_4T1_3 | 3443.93 | 188.446 | 0.990202 | 3284.5 | 0.780929 | 9.12239 | 0.993189 |  |
| STZ_4T1_4 | 3204.75 | 193.131 | 0.988685 | 2987.1 | 0.744593 | 8.59597 | 0.990597 |  |
